# MARKERS OF INFLAMMATION ARE MODIFIED BY SEX, FRAILTY AND EXERCISE IN A HEAD-DOWN TILT BEDREST STUDY

**DOI:** 10.1093/geroni/igae098.3669

**Published:** 2024-12-31

**Authors:** Susan Howlett, Maddison L Hodgins, Judith Godin, Kenneth Rockwood, Scott Grandy

**Affiliations:** Dalhousie University, Halifax, Nova Scotia, Canada; Dalhousie University, Halifax, Nova Scotia, Canada; Nova Scotia Health Authority, Halifax, Nova Scotia, Canada; Dalhousie University, Halifax, Nova Scotia, Canada; Dalhousie University, Halifax, Nova Scotia, Canada

## Abstract

Prolonged bedrest can induce physiological stress thereby exacerbating frailty and inflammation. We explored whether Head-Down Tilt (HDT) bedrest with or without exercise would alter inflammatory markers implicated in frailty. Twenty 55-65-year-old males (baseline frailty index (FI)=0.02±0.02) and females (FI=0.03±0.03) were subjected to HDT bedrest (2-weeks). Half were randomly assigned to a high-intensity interval/aerobic/strength training exercise intervention for 60 minutes/day (Control: n=4 males, 5 females; Exercise n=5 males, 6 females). FI and serum inflammatory cytokines were measured (multiplex assay) during bedrest (days 0,1,3,7,14), and recovery (days 0,4,32). Data were analyzed with linear mixed models. Proinflammatory marker levels (interleukin (IL)-6, IL-8) increased during bedrest in all groups but were higher in males than females (IL-6: ß=8.87, IL-8: ß=12.93; p< 0.01). IL-8 declined during recovery in all groups, although IL-8 and IL-6 remained higher in males (IL-6: ß=9.32, IL-8: ß=2.85, p< 0.01). The anti-inflammatory cytokine IL-10 was higher in males than females during bedrest, declined in recovery and remained highest in males. During bedrest proinflammatory cytokines (IL-1ß, IL-12) increased as frailty increased in all participants (IL- ß: ß=0.01, IL-12: ß=0.02, p< 0.05) whereas monocyte chemoattractant protein-1 levels declined (ß =-20.54, p< 0.05). The anti-inflammatory protein IL-1ra increased with frailty throughout bedrest in all, especially those who exercised (ß=3.15, p< 0.01). In older adults, inflammatory markers increased during bedrest, declined during recovery and were higher in males than females. Some inflammatory markers increased in parallel with frailty during bedrest. Exercise also increased IL-1ra levels, which may help reduce inflammation in older adults during prolonged bedrest.

